# Testing a Recombinant Form of Tetanus Toxoid as a Carrier Protein for Glycoconjugate Vaccines

**DOI:** 10.3390/vaccines11121770

**Published:** 2023-11-28

**Authors:** Davide Oldrini, Roberta Di Benedetto, Martina Carducci, Daniele De Simone, Luisa Massai, Renzo Alfini, Barbara Galli, Brunella Brunelli, Amanda Przedpelski, Joseph T. Barbieri, Omar Rossi, Carlo Giannelli, Rino Rappuoli, Francesco Berti, Francesca Micoli

**Affiliations:** 1 GSK Vaccines Institute for Global Health (GVGH), via Fiorentina 1, 53100 Siena, Italy; davide.x.oldrini@gsk.com (D.O.); roberta.x.di-benedetto@gsk.com (R.D.B.); martina.x.carducci@gsk.com (M.C.); daniele.x.desimone@gsk.com (D.D.S.); luisa.x.massai@gsk.com (L.M.); renzo.x.alfini@gsk.com (R.A.); omar.x.rossi@gsk.com (O.R.); carlo.x.giannelli@gsk.com (C.G.); 2 GSK, via Fiorentina 1, 53100 Siena, Italy; barbara.x.galli@gsk.com (B.G.); brunella.x.brunelli@gsk.com (B.B.); francesco.x.berti@gsk.com (F.B.); 3 Department of Microbiology and Immunology, Medical College of Wisconsin, Milwaukee, WI 53226, USA; ahill@mcw.edu (A.P.); jtb01@mcw.edu (J.T.B.); 4 Fondazione Biotecnopolo, via Fiorentina 1, 53100 Siena, Italy; rino.rappuoli@biotecnopolo.it

**Keywords:** glycoconjugate, tetanus toxoid, carrier protein

## Abstract

Glycoconjugate vaccines play a major role in the prevention of infectious diseases worldwide, with significant impact on global health, enabling the polysaccharides to induce immunogenicity in infants and immunological memory. Tetanus toxoid (TT), a chemically detoxified bacterial toxin, is among the few carrier proteins used in licensed glycoconjugate vaccines. The recombinant full-length 8MTT was engineered in *E. coli* with eight individual amino acid mutations to inactivate three toxin functions. Previous studies in mice showed that 8MTT elicits a strong IgG response, confers protection, and can be used as a carrier protein. Here, we compared 8MTT to traditional carrier proteins TT and cross-reactive material 197 (CRM_197_), using different polysaccharides as models: Group A *Streptococcus* cell-wall carbohydrate (GAC), *Salmonella* Typhi Vi, and *Neisseria meningitidis* serogroups A, C, W, and Y. The persistency of the antibodies induced, the ability of the glycoconjugates to elicit booster response after re-injection at a later time point, the eventual carrier-induced epitopic suppression, and immune interference in multicomponent formulations were also evaluated. Overall, immunogenicity responses obtained with 8MTT glycoconjugates were compared to those obtained with corresponding TT and, in some cases, were higher than those induced by CRM_197_ glycoconjugates. Our results support the use of 8MTT as a good alternative carrier protein for glycoconjugate vaccines, with advantages in terms of manufacturability compared to TT.

## 1. Introduction

Glycoconjugation is a well-established technology for the development of polysaccharide (PS)-based vaccines and, over the last decades, glycoconjugates have demonstrated great impact on global health [1,2,3,4,5].

Chemical conjugation to a carrier protein allows the overcoming of limitations of plain PS vaccines: B-cell–T-cell interaction also makes glycoconjugate vaccines effective in infants, with the ability to elicit a memory response [6,7,8].

Among the variables that can impact the immunogenicity of glycoconjugates [9,10], the carrier protein selected plays a major role. Only five carrier proteins are currently used in licensed glycoconjugate vaccines [3,11]: diphtheria toxoid (DT) and tetanus toxoid (TT), derived from the respective toxins after chemical detoxification [12]; cross-reactive material 197 (CRM_197_), a mutant version of the diphtheria toxin [13]; non-typeable H. influenzae (NTHi) protein D (PD) [14]; and the outer membrane protein complex of serogroup B meningococcus (OMPC) [15,16].

DT and TT were initially selected because of their safety track record established over decades of vaccination against diphtheria and tetanus. In TT and DT manufacturing processes, detoxification is achieved by chemical treatment with formaldehyde, a process that can result in extensive modification and heterogeneity of the corresponding preparations, rendering more difficult product characterization and impacting batch-to-batch consistency [17,18,19]. To avoid this, when possible, genetic detoxification should be preferred, resulting in well-defined protein structures. This was the case for CRM_197_, originally produced from the *Corynebacterium diphtheriae* manufacturing platform [20] and, more recently, produced at higher yields and lower cost through recombinant DNA techniques in *E. coli* (rCRM and EcoCRM) [21,22].

Recently, a recombinant full-length version of TT was produced with eight individual amino acid mutations (8MTT) to inactivate catalysis, light chain translocation, and host receptor-binding functions of tetanus toxin, while retaining 99.4% amino acid identity to the native form [23]. 8MTT was found to be >50 million-fold less toxic in outbred mice than the native toxin. Vaccination with 8MTT elicited a strong IgG response in mice and conferred protection against the toxin in a challenge mouse model [23]. Moreover, with 8MTT, detoxification by chemical treatment with formaldehyde is avoided, leading to higher consistency of production and easier characterization of the well-defined protein.

8MTT has already been produced at gram scale in *E. coli* and preliminary data have been generated by testing it as carrier, using the tick peptide P0 (which is under consideration as an immunogen for a cattle tick fever vaccine) and the capsular polysaccharide of Haemophilus influenzae type b (PRP) as antigens [24].

In this study, we evaluated 8MTT as a carrier for glycoconjugates and compared it to more traditional carrier proteins such as CRM_197_ and TT. Different polysaccharides were used as models: Group A *Streptococcus* cell-wall carbohydrate (GAC) [25], *Salmonella* Typhi Vi [26,27], and *Neisseria meningitidis* (Men) serotypes A, C, W, and Y oligosaccharides (OSs) [28,29]. Two common interference mechanisms observed following vaccination with glycoconjugate vaccines are: (1) carrier priming (and suppression), whereby preexisting immunity to a carrier protein impacts subsequent responses to a hapten/saccharide linked to the same carrier [30,31]; (2) bystander interference, whereby coadministration and/or combinations of vaccines containing a given conjugated protein induce interference that extends to unrelated antigens that are part of the combinations in use [30,32,33]. Here, we evaluated the carrier-priming effect of 8MTT in comparison to that of the other protein carriers [34], together with the potential immuno-interference generated by combining multiple Men glycoconjugates sharing the same carrier protein. The results from this work confirmed the possibility of using 8MTT as a valid alternative carrier protein for future glycoconjugate vaccines.

## 2. Materials and Methods

### 2.1. Polysaccharides

GAC was purified, as previously described [35], from the GAS51∆M1 strain, an M protein mutant of HRO-K-51, which was kindly provided by the University of Rostock. Vi was obtained from *Citrobacter* NVGH 328 isolate, according to the procedure previously described [36]. MenA-, MenC-, MenW-, and MenY-activated OSs were obtained from GSK R&D (Siena, Italy).

### 2.2. Proteins

CRM_197_ and TT carrier proteins were kindly provided by GSK R&D (Siena, Italy). Recombinant 8MTT was produced and purified at the Medical College of Wisconsin, as previously described [23].

### 2.3. Glycoconjgates Synthesis

#### 2.3.1. GAC Glycoconjugates

GAC was glycoconjugated to all carrier proteins (CRM_197_, TT, and 8MTT), as previously described for CRM_197_ carrier protein [35]. Briefly, GAC at ~2 mg/mL was oxidized with 8 mM NaIO_4_ in phosphate buffer at pH 7.2, at 25 °C in the dark for 30 min. After that, NaIO_4_ excess was quenched with 16 mM Na_2_SO_3_ at room temperature (RT) for 15 min, keeping the reaction mixture in mild agitation. Oxidized GAC (GACox) was then exchanged in water through tangential flow filtration (TFF) with a 10 kDa cut-off membrane Sartorius Hydrosart (200 cm^2^ membrane area). Lyophilized GAC at 40 mg/mL was glycoconjugated to the proteins using a GACox-to-protein 1:1 *w*/*w* ratio, in the presence of NaBH_3_CN 5 mg/mL, in borate buffer at pH 8. The conjugation mixture was mixed at 37 °C for 4 h. Then, residual unreacted aldehydes of GACox were quenched, adding NaBH_4_ (NaBH_4_:GACox *w*/*w* ratio of 0.5 to 1) and incubating at 37 °C for 2 h. CRM_197_ glycoconjugate was purified via TFF, using a membrane with a cut-off of 50 kDa molecular weight, while 8MTT and TT glycoconjugates were purified by Amicon Ultra 30 kDa cut-off.

#### 2.3.2. Vi Glycoconjugates

Vi was glycoconjugated to all carrier proteins (CRM_197_, TT, and 8MTT), as previously described for CRM_197_ [36]. In particular, to a solution of Vi at 4.2 mg/mL in 100 mM MES pH 6, N-hydroxysuccinimide (NHS) and, then, 1-Ethyl-3-(3-dimethylaminopropyl)carbodiimide (EDAC) were added, to reach a 0.1 M NHS and EDAC/Vi repeating-units molar ratio of 5. The proteins were derivatized with adipic acid dihydrazide (ADH) and the number of ADH linkers per protein was measured through a 2,4,6-trinitrobenzene 1-sulfonic acid (TNBS) assay, resulting in 5 for CRM_197_, 24 for TT, and 20 for 8MTT. The reaction for Vi activation was mixed at RT for 1 h and the proteins previously derivatized with ADH were added with a protein/Vi ratio 1:1 *w*/*w*, reaching a Vi concentration of 3.3 mg/mL in 20 mM MES at pH 6, and mixed for 2 h at RT. Vi glycoconjugates were purified by size-exclusion chromatography on a 1.6 cm × 90 cm Sephacryl S1000 column (Cytiva Life Sciences, Marlborough, MA, USA; formerly GE Healthcare Life Sciences) eluted at 0.5 mL/min in PBS. Fractions at higher molecular weight that did not overlap with free PS were collected.

#### 2.3.3. Men Glycoconjugates

MenA, MenC, MenW, and MenY OSs (average size of 4.5 kDa) were glycoconjugated to TT and 8MTT. Briefly, lyophilized MenACWY-activated OSs were glycoconjugated to the carrier proteins at 20 mg/mL in PBS pH 7.2 using an OS-to-protein 25:1 mol active esters:mol carrier protein ratio. Conjugation mixtures were gently mixed overnight at RT, followed by purification with an HiTrap 5 mL column (Cytiva Life Sciences, Marlborough, MA, USA; formerly GE Healthcare Life Sciences). MenA, MenC, MenW, and MenY glycoconjugates to CRM_197_ were provided by GSK R&D (Siena, Italy).

### 2.4. Glycoconjugates Characterization

Micro BCA (Thermo Scientific, Waltham, MA, USA) and HPAEC-PAD [35,36,37] were used to estimate total protein and total PS content, respectively, and to calculate the saccharide-to-protein ratios. Free PS in GAC glycoconjugates was quantified by HPAEC-PAD after separation through DOC precipitation [38], while free Vi was separated by Capto Adhere resin and quantified by HPAEC-PAD [36]. Free OSs in Men glycoconjugates were separated by solid phase extraction (SPE) and quantified by HPAEC-PAD [37]. Glycoconjugates formation was verified by HPLC-SEC and SDS-PAGE analyses, comparing the glycoconjugates with corresponding unconjugated proteins. Glycoconjugates were also tested by differential scanning fluorimetry (DSF) in comparison to the corresponding carrier protein alone by measuring the folding state transition of the protein through the fluorescence intensity ratio at 330 nm and 350 nm as a function of temperature at same concentration and in PBS buffer.

### 2.5. Immunogenicity Studies in Mice and Assessment of Antibody Responses

GAC and Vi glycoconjugates in vivo studies were performed at the GSK Animal Facility (Siena, Italy), in compliance with the relevant guidelines (Italian D.Lgs. n. 26/14 and European directive 2010/63/EU) and the institutional policies of GSK. The animal protocols were approved by the Italian Ministry of Health (AEC project No. 526/2020-PR, approval date 26/05/2020). CD1 mice (8 per group, female, 5 weeks old) were injected intraperitoneally (i.p.) at study days 0 and 28 with 200 µL of antigens. Sera were collected at day −1 (pooled) and at day 27 (single), with a final bleed at day 42 (single sera). In a different scheme, mice were vaccinated at days 0, 28, and 98 and sera were collected at day −1 (pooled) and days 27, 42, and 97 (single), with a final bleed at day 112 (single sera) (Figure S1).

Meningococcal glycoconjugates in vivo studies were performed at Charles River Laboratories (France), accordingly to the European Directive 63/2010. Balb/cByJ mice (8 per group, female, 4–6 weeks old) were vaccinated subcutaneously (s.c.) with 200 µL of antigens at study days 0, 14, and 28. Sera were collected at day −1 (pooled) and at day 27 (single), with a final bleed at day 42 (single sera) (Figure S1).

GAC glycoconjugates were formulated on Alhydrogel (0.7–2 mg/mL Al^3+^) and their adsorption (>90%) was evaluated on formulation supernatants via SDS-PAGE with silver staining detection, following the manufacturer’s instruction (SilverQuest Silver Staining kit, ThermoFisher Scientific, Waltham, MA, USA), after adjuvant removal by two sequential centrifugations (18,000 rcf, 15 min, 4 °C). Vi glycoconjugates were tested not-adjuvanted. Men glycoconjugates were formulated with Alum Phosphate (0.6 mg/mL Al^3+^).

Mouse sera were analyzed for anti-GAC, anti-Vi, anti-MenA, anti-TT, and anti-CRM_197_ total IgG by enzyme-linked immunosorbent assay (ELISA), using GAC-HSA (at the protein concentration of 1 µg/mL in carbonate buffer pH 9.6), Vi (at the concentration of 1 µg/mL in phosphate buffer), MenA PS (at the concentration of 5 µg /mL in PBS pH 8.2), TT (at the concentration of 1 µg /mL in phosphate buffer), and CRM_197_ (at the concentration of 2 µg/mL in carbonate buffer) as coating antigens [35,36].

Human serum bactericidal activity (SBA) against MenA was tested using 25% exogenous human sera as the source of complement (hSBA), derived from donors with no detectable intrinsic bactericidal activity. The human complement source used in the study was obtained according to good clinical practice and the Declaration of Helsinki. Patients provided their written consent (MENB REC 2ND GEN-074 (V72_92) [39].

### 2.6. Statistics

Statistical analysis was performed using GraphPad Prism 7. The immune responses induced by two different formulations were compared by the Mann–Whitney two-tailed test, while the comparison among more than two groups was done by the Kruskal–Wallis test with Dunn’s post hoc analysis. Responses induced by the same formulation at different timepoints were compared through the Wilcoxon matched-pairs signed rank two-tailed test.

Dose-response curve comparisons were performed using CombiStats Software 7.0 (EDQM, France). The linearity and the parallelism of the lines were confirmed. The result of the analysis was reported as a potency value %, with a 95% confidence interval.

## 3. Results

### 3.1. Glycoconjugates Preparation

**Figure 1:**
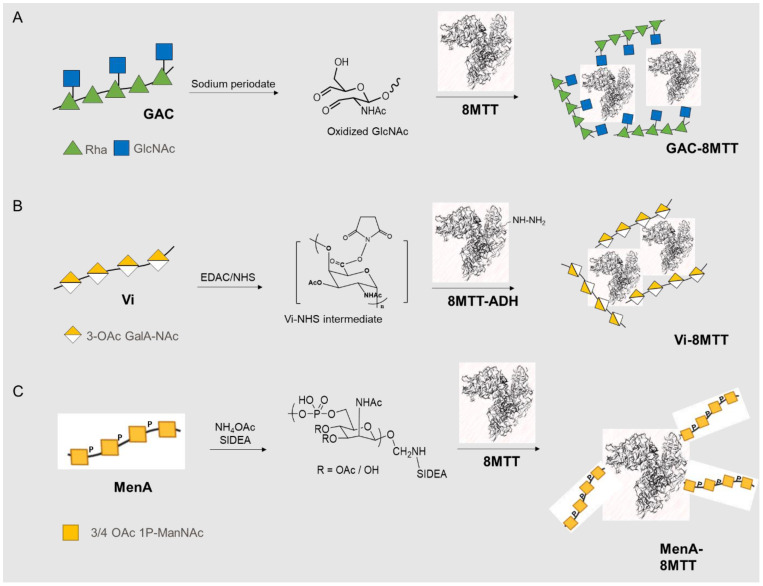
Strategies to produce 8MTT glycoconjugates: (**A**) aldehyde groups randomly generated through oxidization of GAC were linked to carrier protein lysines by reductive amination; (**B**) protein derivatization with ADH and conjugation to Vi PS by carbodiimide chemistry; (**C**) MenA OS (as an example of all Men OSs) terminally activated with SIDEA and linked to lysines of the carrier protein.

Different PS were glycoconjugated to 8MTT, making use of different chemistries (Figure 1). Analogous approaches were used for linkage of the PS to the benchmark carrier proteins CRM_197_ and chemically detoxified TT. In particular, GlcNAc residues on GAC were randomly oxidized with sodium periodate to generate aldehydic groups that were subsequently linked to lysines on the carrier protein through reductive amination (Figure 1A) [35]. Vi was instead glycoconjugated through carbodiimide chemistry to the protein previously derivatized with adipic acid dihydrazide (ADH) (Figure 1B) [36,40]. Meningococcal OSs were activated via reductive amination with ammonium acetate for coupling to a bis-N-hydroxysuccinimidyl adipate linker (SIDEA). The active ester group introduced was finally glycoconjugated to the carrier protein (Figure 1C).

**Figure 2:**
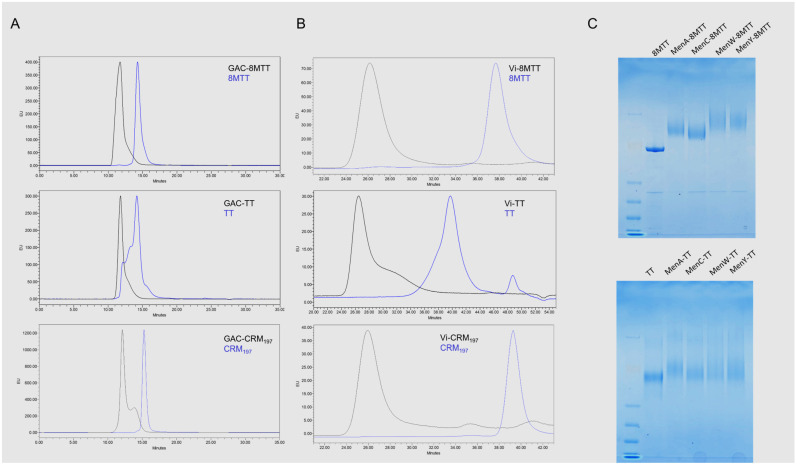
Glycoconjugates characterization by HPLC-SEC (fluorescence emission detection) of GAC conjugates (**A**) and Vi conjugates (**B**), in comparison to corresponding unconjugated proteins; 80 µL of sample injected on a TSK gel G3000 PWXL column (**A**) or TSK gel G6000-5000 PW columns connected in series (**B**); 0.1 M NaCl, 0.1 M NaH_2_PO_4_, 5% CH_3_CN pH 7.2 at 0.5 mL/min. Characterization by SDS-PAGE analysis (7% Tris-acetate gel) of the Men glycoconjugates in comparison to unconjugated proteins (**C**). 3 µg of glycoconjugated protein and 3 µg of unconjugated ones were loaded per well.

Glycoconjugates formation was verified by high-performance liquid chromatography–size-exclusion chromatography (HPLC-SEC) (for GAC and Vi glycoconjugates) or sodium dodecyl sulfate-polyacrylamide gel electrophoresis (SDS-page) (for Men glycoconjugates) (Figure 2). For all PS and with all proteins used, no free protein was detected in the conjugation mixtures at the end of the reaction time, while unconjugated saccharides were removed using different purification methods, according to the glycoconjugated PS. Independently from the carrier protein used, all GAC and Vi glycoconjugates showed a similar PS-to-protein weight ratio, in the ranges of 0.37–0.51 for GAC glycoconjugates and 0.42–0.54 for Vi glycoconjugates. For Men glycoconjgates, PS-to-protein ratios are reported in Table S1. Free saccharide was <2% for GAC glycoconjugates and <20% for Vi and Men glycoconjugates. The main characteristics of all generated glycoconjugates are reported in Figure 2 and Table S1.

### 3.2. Immunogenicity Studies in Mice with GAC and Vi Glycoconjugates, Comparing 8MTT to CRM_197_ and TT Carrier Proteins

**Figure 3:**
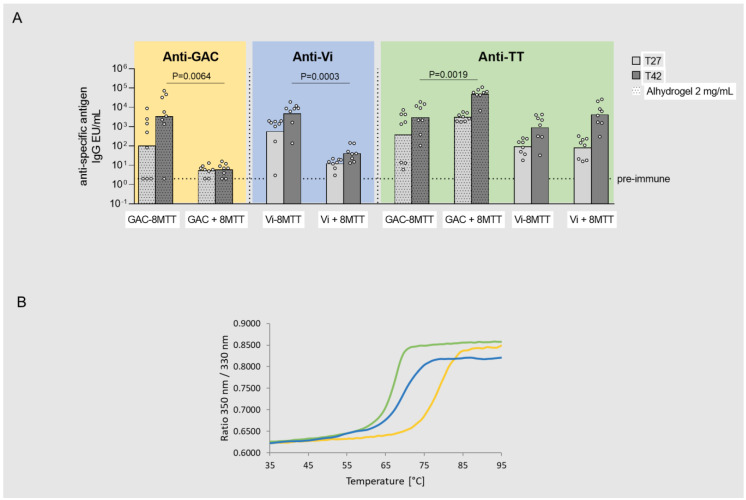
(**A**) Immunogenicity of GAC and Vi physically mixed (GAC + 8MTT and Vi + 8MTT) or glycoconjugated to 8MTT (GAC−8MTT and Vi−8MTT). CD1 mice were immunized intraperitoneally (i.p.) at days 0 and 28 with 1.5 µg GAC/dose or 1.0 µg Vi/dose, and 3.2 µg or 1.9 µg 8MTT, respectively. GAC groups were all formulated with 2 mg/mL Alhydrogel. Sera collected at days 27 (T27) and 42 (T42) were analyzed by enzyme-linked immunosorbent assay (ELISA), using GAC−HSA, Vi and TT as coating antigens. Individual anti-antigen specific IgG EU/mL are reported as dots, with bars corresponding to groups’ geometric means. (**B**) Differential scanning fluorimetry (DSF) F330/350 fluorescence ratio intensity of 8MTT (green line), GAC−8MTT (yellow line), and Vi-8MTT (blue line) plotted against temperature.

GAC and Vi glycoconjugated to 8MTT were compared in an in vivo study in mice to the corresponding physical mixtures, confirming that covalent linkage between the saccharide and 8MTT is needed to elicit a significant anti-PS IgG response. Both glycoconjugates elicited significantly higher anti-GAC and Vi IgG responses than those of the relative physical mixtures, both 4 weeks after the first injection and 2 weeks after the second injection (Figure 3A).

Anti-TT IgG responses were also assessed using TT as the coating antigen. Only TT was used, based on previous data showing the comparability of ELISA results using TT or 8MTT as coating antigens [24]. A decline in the anti-TT IgG response induced by 8MTT was evidenced after conjugation to both GAC and Vi, with statistical significance only for GAC (Figure 3A).

Through a differential scanning fluorimetry (DSF) analysis [41], the impact of conjugation of GAC and Vi on the 8MTT thermal-stability profile was verified. This indicated partial modification of the protein after conjugation, which could justify a different anti-TT IgG response elicited by the glycoconjugates with respect to the unconjugated proteins. Indeed, for GAC, the differences between unconjugated and glycoconjugated proteins detected by DSF resulted more prominent with respect to Vi, corresponding to a significant reduction in the anti-TT IgG response (Figure 3B).

GAC and Vi glycoconjugates were tested in mice to compare 8MTT with more traditional carrier proteins (e.g., TT and CRM_197_). In particular, both GAC and Vi glycoconjugates were tested at three different PS doses, ranging from 1.5 to 0.166 µg of GAC and from 1.0 to 0.1 µg of Vi. GAC glycoconjugates were tested both with Alhydrogel (at all the doses) and without Alhydrogel (only at the higher dose) [35]. Vi glycoconjugates were, instead, tested only in the absence of Alhydrogel [36].

**Figure 4:**
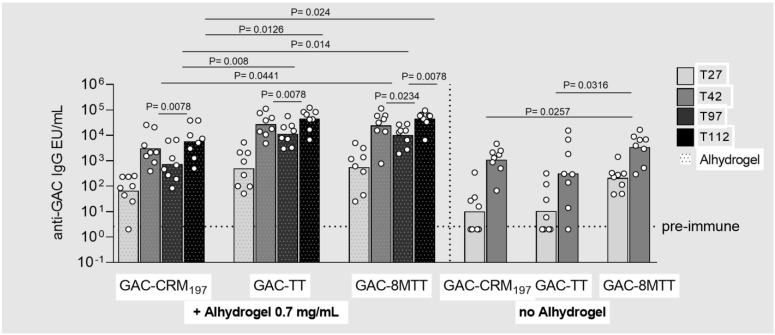
Immunogenicity of GAC glycoconjugated to 8MTT (GAC−8MTT), TT (GAC−TT), or CRM_197_ (GAC−CRM_197_). CD1 mice were immunized intraperitoneally (i.p.) at days 0 and 28 with 1.5 µg GAC/dose (corresponding to 3.2 µg of 8MTT, 4.0 µg of TT and 2.9 µg of CRM_197_) in the presence or absence of 0.7 mg/mL Alhydrogel (Al^3+^). A third immunization at day 98 was performed only in the presence of Alhydrogel. Sera collected at days 27 (T27), 42 (T42), 97 (T97), and 112 (T112) were analyzed by ELISA. Individual anti-GAC IgG EU/mL are reported as dots, with bars corresponding to groups’ geometric means.

Independently from the carrier protein used, no dose response was observed for GAC glycoconjugates when formulated with Alhydrogel. All GAC glycoconjugates induced similar anti-GAC IgG response at the three different GAC doses tested (Figure S2). Only at the higher dose of 1.5 µg GAC did GAC-8MTT elicit a significantly higher anti-GAC IgG response than that of GAC-CRM_197_, two weeks after the second injection (Figure 4).

Interestingly, in the absence of Alhydrogel, GAC-8MTT elicited a significantly higher anti-GAC IgG response 27 days after one injection than the other two glycoconjugates, for which 50% of the animals showed no response. The responses elicited by all glycoconjugates became similar 14 days after the second injection (Figure 4).

In the presence of Alhydrogel, we also evaluated the longevity of the immune response at day 97, 70 days after the second injection. For all the glycoconjugates, the anti-GAC IgG response significantly decreased from day 42 to day 97, with similar decay rates. Finally, the ability of the different glycoconjugates to boost the response at a later time point was assessed. Interestingly, only 8MTT glycoconjugate significantly boosted the response two weeks after the third injection at day 98. At day 97 and at day 112, the 8MTT and TT glycoconjugates elicited higher responses than the CRM_197_ glycoconjugate (Figure 4).

**Figure 5:**
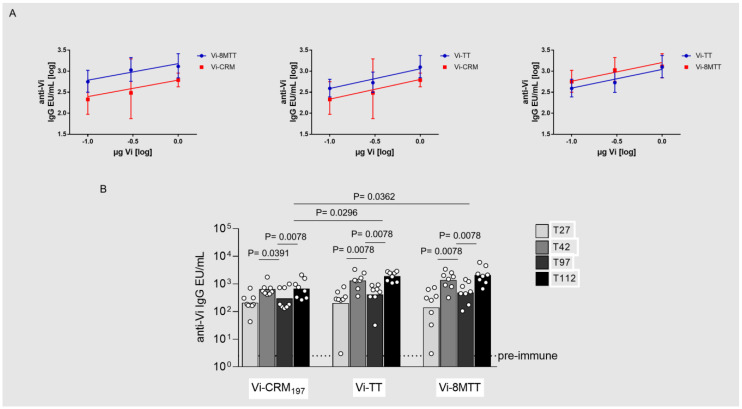
Immunogenicity in mice of Vi glycoconjugated to 8MTT (Vi−8MTT), TT (Vi−TT), or CRM_197_ (Vi−CRM_197_). CD1 mice were immunized intraperitoneally (i.p.) at days 0 and 28 with 1.0 µg Vi/dose (corresponding to 1.9 µg of 8MTT, 2.0 µg of TT, and 2.4 µg of CRM_197_) without Alhydrogel. (**A**) Parallel line analysis showing dose-response curves at day 42 of the different formulations (log transformed IgG EU/mL titers in Y axis and log transformed Vi doses in X axis); (**B**) a third immunization was performed at day 98. Sera collected at days 27 (T27), 42 (T42), 97 (T97), and 112 (T112) were analyzed by ELISA. Individual anti-Vi IgG EU/mL are reported as dots, with bars corresponding to groups’ geometric means.

In the case of Vi, at day 27, a dose response was observed only for the CRM_197_ glycoconjugate. At this timepoint, similar anti-Vi IgG responses were elicited by all the glycoconjugates at each dose tested (Figure S3). Two weeks after the second immunization, significant dose-response relationships were instead verified for all the glycoconjugates. Comparing the dose-response curves, Vi-CRM_197_ induced a significantly lower response than Vi-8MTT (with a potency of 11.2%; 95% confidence interval 0–48.4%), while similar responses were elicited comparing Vi-CRM_197_ to Vi-TT (potency of 33.5%; 95% confidence interval 3–100.8%) and Vi-TT to Vi-8MTT (potency of 44.4%; 95% confidence interval 10.8–108.2%) (Figure 5A).

Additionally, at 1 µg of Vi/dose, we evaluated the longevity of the immune response 70 days after the second immunization. For all conjugates, the anti-Vi IgG response significantly decreased from day 42 to day 97, with similar decay rates. The ability of the different glycoconjugates to boost the response at a later time point was assessed and all glycoconjugates boosted the response two weeks after the third injection at day 98. Higher anti-Vi IgG responses were induced by 8MTT and TT glycoconjugates than by CRM_197_ glycoconjugate two weeks after the third injection (Figure 5B).

### 3.3. Carrier-Priming Effect on the Immune Response Elicited in Mice by MenA Glycoconjugates

The carrier-priming effect was evaluated on the anti-MenA response elicited by MenA glycoconjugates after priming with two injections of the homologous carrier proteins (8MTT, TT or CRM_197_). The doses used and the immunization scheme were selected based on a previous study in mice [34].

**Figure 6:**
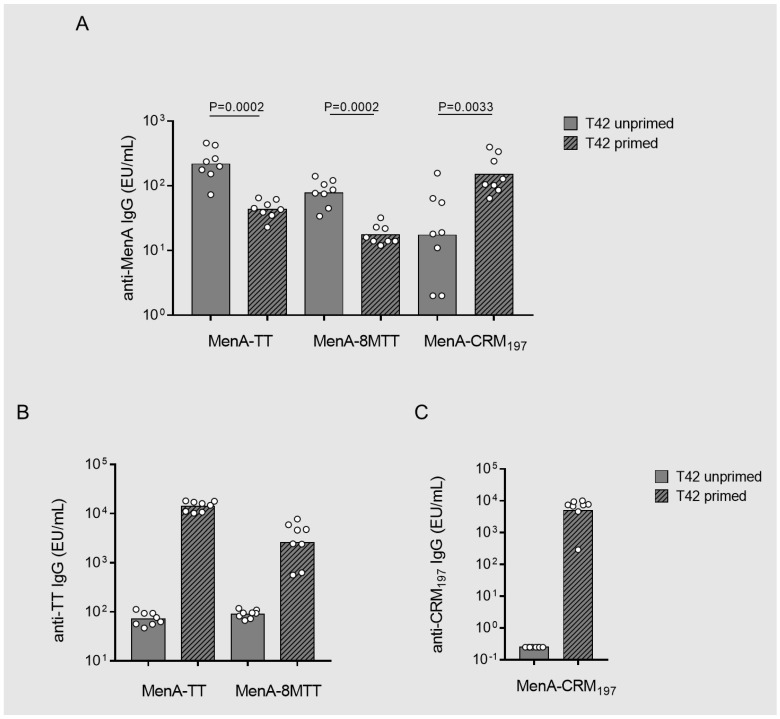
Immunogenicity in mice of MenA glycoconjugated to 8MTT (MenA−8MTT), TT (MenA−TT), or CRM_197_ (MenA−CRM_197_). Balb/c mice were immunized subcutaneously (s.c). All groups received 50 µg of the carrier proteins (primed groups, line pattern) or Alum phosphate at 0.6 mg/mL (unprimed groups, no pattern) at days 0 and 14 and 2.0 µg of corresponding MenA glycoconjugates (corresponding to 8 µg of 8MTT, 6.9 µg of TT, and 5 µg of CRM_197_) at day 28 with Alum phosphate at 0.6 mg/mL as adjuvant. Sera were collected at day 42 (T42). Summary graphs of anti-MenA (**A**), anti−TT (**B**), and anti−CRM_197_ (**C**) specific IgG geometric mean units (bars) and individual antibody levels (dots) are reported.

After priming with TT or 8MTT proteins, corresponding MenA glycoconjugates elicited a significantly lower anti-MenA IgG response than the response induced by not-primed MenA-TT or MenA-8MTT. An opposite effect was, instead, confirmed for CRM_197_ [34]. When MenA-CRM_197_ was primed with the corresponding protein, the anti-MenA IgG response was significantly enhanced compared to the response elicited by not-primed MenA-CRM_197_ (Figure 6A).

**Figure 7:**
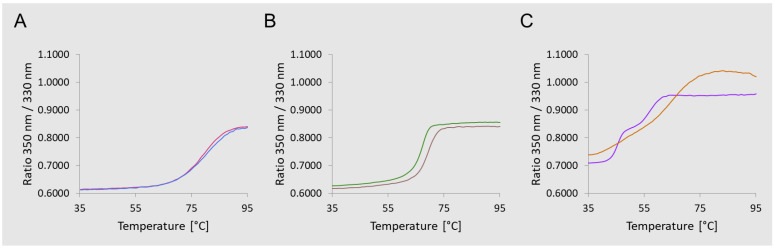
Differential scanning fluorimetry (DSF) F330/350 fluorescence ratio intensity of (**A**) TT (red line) vs. MenA−TT (blue line); (**B**) 8MTT (green line) vs. MenA−8MTT (brown line); (**C**) CRM_197_ (violet line) vs. MenA−CRM_197_ (orange line), plotted against temperature.

Conformational changes before and after conjugation were observed and were likely the main driver of the differences in the carrier-priming effect [34]. DSF analysis was carried out to compare TT, 8MTT, and CRM197 and the corresponding MenA glycoconjugates, revealing that for TT and 8MTT, protein stability and folding were not impacted by the conjugation, while CRM_197_ folding was strongly impacted by MenA linkage (Figure 7).

The generation of anti-TT antibodies with priming doses of the proteins alone (Figure 6B) suppresses the response against the corresponding glycoconjugates. However, comparing anti-MenA IgG induced by 8MTT-primed MenA-8MTT and TT-primed MenA-8MTT, 8MTT had a lower carrier-priming suppression effect than that of chemically detoxified TT (Figure 6A).

### 3.4. Immunointerference on Anti-MenA Response in MenACWY Tetravalent Formulations by Using 8MTT, TT or CRM_197_ as Carrier Proteins

MenACWY 4-component formulations were compared in an immunogenicity study in mice, with corresponding MenA monovalent formulations.

**Figure 8:**
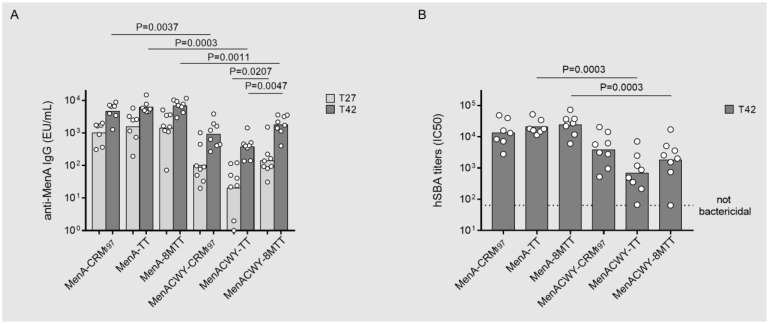
Immunogenicity in mice of MenA glycoconjugated to 8MTT (MenA−8MTT), TT (MenA−TT), or CRM_197_ (MenA−CRM_197_) and MenACWY corresponding tetravalent formulations. Balb/c mice were immunized subcutaneously (s.c) at days 0, 14 and 28 with 2.0 µg MenA/doses and 1 µg MenCWY/doses with Alum phosphate at 0.6 mg/mL. Sera collected at days 27 (T27) and 42 (T42) were analyzed by ELISA using MenA PS as the coating antigen (**A**) and by serum bactericidal assay using human sera as the source of complement (hSBA) only at day 42 (T42) (**B**). Geometric mean units (bars) and individual antibody levels (dots) are reported.

Comparing the anti-MenA IgG response induced by each monovalent MenA glycoconjugate formulation with the corresponding tetravalent formulation (MenA-CRM_197_ vs. MenACWY-CRM_197_; MenA-TT vs. MenACWY-TT, and MenA-8MTT vs. MenACWY-8MTT), independently from the protein, the monovalent glycoconjugate induced a significantly higher anti-MenA IgG response than the corresponding 4-valent formulation (Figure 8A). Data generated indicated that for all tested carrier proteins, the anti-MenA IgG response was negatively impacted by immunological interference when combined in multivalent formulations. However, the suppression was inferior for the combination that included MenA-8MTT in comparison with the combination that included MenA-TT, both post-second and post-third immunization. The antibodies elicited by MenA-8MTT were statistically higher than the antibodies induced by MenA-TT. The results were confirmed by examining the bactericidal activity of the antibodies induced (Figure 8B).

## 4. Discussion

The nature of the carrier protein is one of the main parameters that can affect the immune response elicited by glycoconjugate vaccines. So far, licensed glycoconjugate vaccines against bacterial infections have made use of few carrier proteins. Among these is TT, which is obtained through chemical detoxification of the corresponding toxin [42].

Recently, a recombinant form of TT has been produced: no toxicity of 8MTT has been confirmed and the ability of the protein to elicit a strong immune response and protection against TT challenge ha been verified [23]. Compared to TT, 8MTT is easier, faster, and safer to manufacture, with higher purity and batch-to-batch consistency. Here, we verified that 8MTT is more suitable for conjugation to PS than TT. Indeed, TT is often purified prior to conjugation to obtain a monomeric fraction, because of the presence of aggregates and low purity. The 8MTT is, instead, already produced as a well-defined pure monomer that is ready for conjugation [24]. When TT is used without prior purification, the separation of unconjugated TT from the glycoconjugate can be more difficult to achieve, as verified here for GAC and Men oligosaccharides, and the glycoconjugate could contain fractions of unconjugated TT at a higher molecular weight (Figure 2).

Here, the 8MTT was compared to CRM_197_ and TT carrier proteins for glycoconjugate vaccines, and all different chemistries tested, according to the different PS used (Group A *Streptococcus* cell-wall carbohydrate (GAC) [35], *Salmonella* Typhi Vi, and *Neisseria meningitidis* (Men) serotypes A, C, W, and Y OSs), worked well with 8MTT.

GAC-, Vi- and MenA-8MTT glycoconjugates induced similar immunogenicity to that of corresponding TT glycoconjugates and, in some cases, higher than that induced by CRM_197_ glycoconjugates. Also, both for Vi and GAC, 8MTT glycoconjugates boosted the responses after immunization at a later time point, and also elicited a significant response post one injection in the absence of aluminum as adjuvant.

Other studies showed no impact of the carrier protein on the immune response elicited by Vi (CRM_197_, DT, TT, *Pseudomonas aeruginosa* exotoxin A (rEPA)) [40,43], or MenA (CRM_197_, DT, TT) [34] glycoconjugates. To our knowledge, this is the first study to directly compare the impact of the commonly used carriers CRM_197_ and TT on the immunogenicity elicited by GAC glycoconjugates.

Immune interference resulting in a decrease in the anti-PS immune response has been, in some cases, reported after pre- or co-exposure to commonly used carrier proteins, thus driving to the need to identify new carrier proteins [3]. For this reason, these effects were studied here for 8MTT. We found that, despite physical differences between 8MTT and TT, the behavior of these two proteins was very similar and probably associated with the major rigidity of the proteins and the ability to conserve the original structure and the conformation after glycoconjugation, as verified here by DSF analysis. It would be interesting to further explore this aspect and to verify how previous anti-TT vaccination may have an impact on the efficacy of glycoconjugate vaccines based on 8MTT. Differently from TT and 8MTT, priming with CRM_197_ resulted in an enhanced anti-MenA IgG response, confirming results previously published [26]. Due to stronger conformational changes after glycoconjugation, glycoconjugate recognition by anti-carrier antibodies should be limited, facilitating glycoconjugate capture by antigen-presenting cells and the expansion of carrier-specific B cells [44,45].

The anti-MenA IgG response induced by tetravalent vaccine formulations was inferior to that of the monovalent vaccine formulations, independently from the carrier protein used. However, the suppression was inferior for the combination including MenA-8MTT, in comparison with MenA-TT. The use of 8MTT protein might partially overcome the carrier protein-mediated interference within multivalent PS glycoconjugate vaccines, as was also observed for pneumococcal vaccines [46].

More data will need to be collected in terms of the quality and functionality of the response elicited by 8MTT glycoconjugates but, overall, our data confirm the possibility of using 8MTT as a good alternative carrier protein for glycoconjugate vaccines, with advantages in terms of manufacturability and similar anti-polysaccharide specific IgG responses to TT.

## Data Availability

The authors declare that data are contained within the article and in the Supplementary Materials.

## Supplementary Materials

The following supporting information can be downloaded at: https://www.mdpi.com/article/10.3390/vaccines11121770/s1. GSK is committed to the replacement, reduction, and refinement of animal studies (3Rs). Non-animal models and alternative technologies are part of our strategy and employed where possible. When animals are required, application of robust study design principles and peer review minimizes animal use, reduces harm, and improves benefit in studies.
