# Supplementary material for: Testing a Recombinant Form of Tetanus Toxoid as a Carrier Protein for Glycoconjugate Vaccines
Source: Vaccines (Basel). 2023 Nov 28;11(12):1770. doi: 10.3390/vaccines11121770 (PMC10747096; doi:10.3390/vaccines11121770)
Supplement: Supplementary file 1 [file vaccines-11-01770-s001.zip › vaccines-2648606 supplementary.pdf]

vaccines

MDPI

Supplemetary Material

Testing a Recombinant Form of Tetanus Toxoid as a Carrier Protein for Glycoconjugate Vaccines

Davide Oldrini [1,†], Roberta Di Benedetto [1,†], Martina Carducci [1], Daniele De Simone [1], Luisa Massai [1], Renzo Alfini [1], Barbara Galli [2], Brunella Brunelli [2], Amanda Przedpelski [3], Joseph T. Barbieri [3], Omar Rossi [1], Carlo Giannelli [1], Rino Rappuoli [4], Francesco Berti [2] and Francesca Micoli [1,*]

1 GSK Vaccines Institute for Global Health (GVGH), via Fiorentina 1, 53100 Siena, Italy; davide.x.oldrini@gsk.com (D.O.); roberta.x.di-benedetto@gsk.com (R.D.B.); martina.x.carducci@gsk.com (M.C.); daniele.x.desimone@gsk.com (D.D.S.); luisa.x.massai@gsk.com (L.M.); renzo.x.alfini@gsk.com (R.A.); omar.x.rossi@gsk.com (O.R.); carlo.x.giannelli@gsk.com (C.G.);
2 GSK, via Fiorentina 1, 53100 Siena, Italy; barbara.x.galli@gsk.com (B.G.); brunella.x.brunelli@gsk.com (B.B.); francesco.x.berti@gsk.com (F.B.)
3 Department of Microbiology and Immunology, Medical College of Wisconsin, Milwaukee, WI 53226, USA; ahill@mcw.edu (A.P.); jtb01@mcw.edu (J.T.B.)
4 Fondazione Biotecnopolo, via Fiorentina 1, 53100 Siena, Italy; rino.rappuoli@biotecnopolo.it
* Correspondence: francesca.x.micoli@gsk.com
† These authors contributed equally to this work and share first authorship.

Table S1. List of synthesized 8MTT and benchmark glycoconjugates tested in mice: glycoconjugation chemistry used and total PS to protein ratio.

Conjugate	Chemistry used	Total PS/Protein w/w ratio
GAC-CRM$_{197}$	Random PS oxidation + reductive amination	0.51
GAC-TT		0.37
GAC-8MTT		0.47
Vi-CRM$_{197}$	Protein derivatization with ADH + random linkage to Vi through carbodiimide chemistry	0.42
Vi-TT		0.49
Vi-8MTT		0.54

Conjugate	Chemistry used	Total PS/Protein w/w ratio	Total PS/Protein mol/mol ratio
MenA-CRM$_{197}$	Terminal saccharide activation with NH2 and SIDEA	0.4	6.5
MenA-TT		0.29	12.0
MenA-8MTT		0.25	10.4
MenC-CRM$_{197}$		0.57	6.0
MenC-TT		0.13	3.5
MenC-8MTT		0.16	4.3
MenW-CRM$_{197}$		0.67	5.8
MenW-TT		0.15	3.3

MenW-8MTT	0.08	1.8
MenY-CRM197	0.62	5.4
MenY-TT	0.15	3.3
MenY-8MTT	0.15	3.3

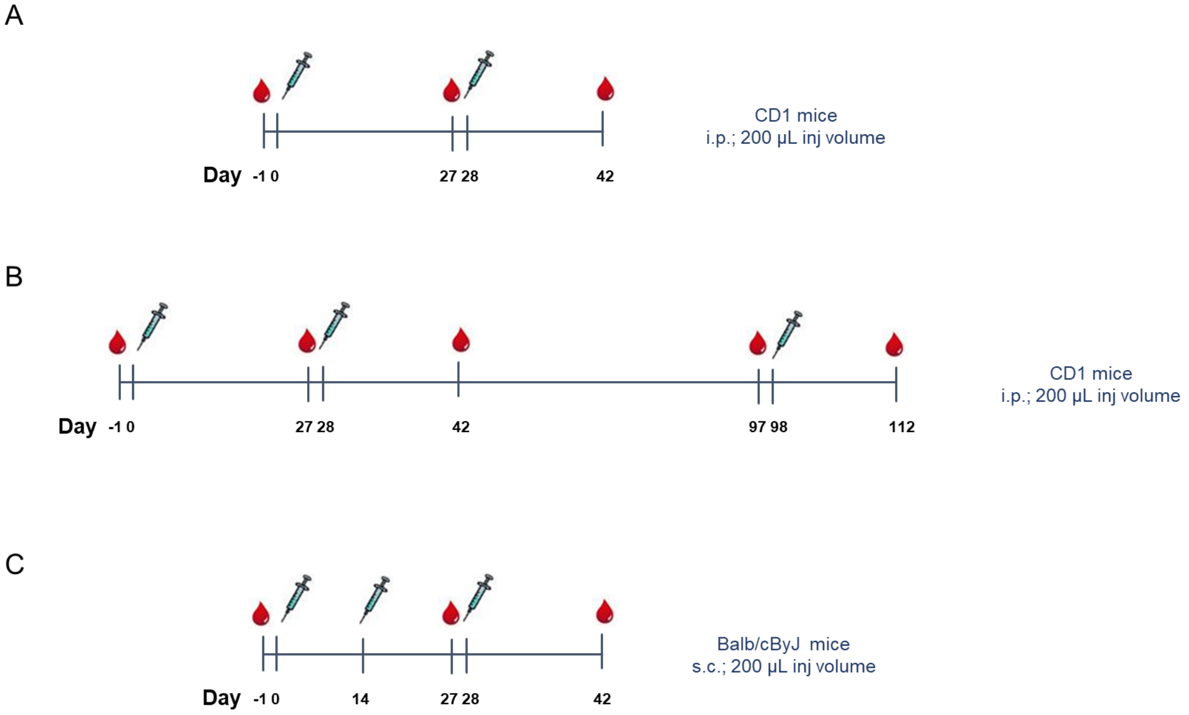

Figure S1. Immunogenicity study designs for GAC, Vi (A, B) and meningococcal glycoconjugates (C).

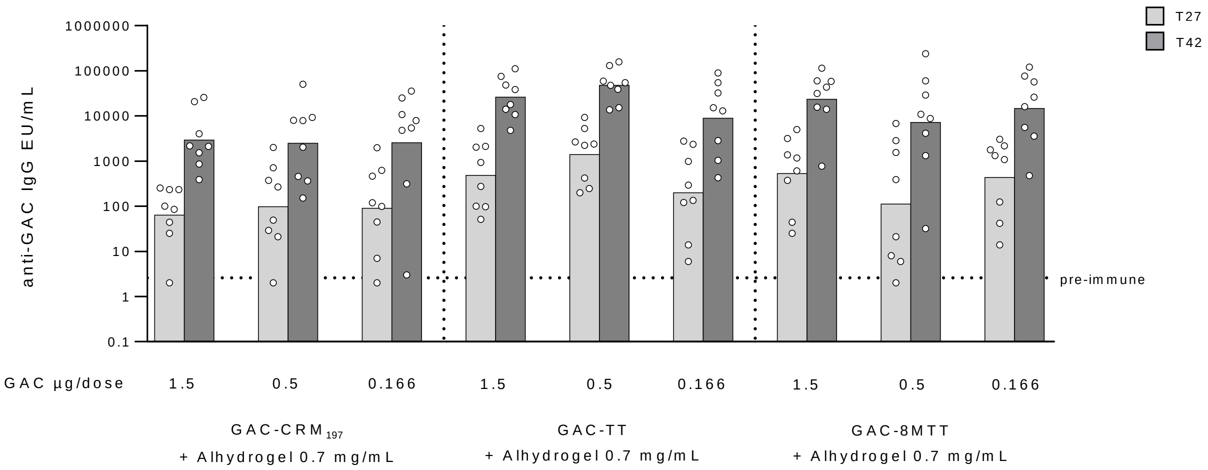

Figure S2. Immunogenicity in mice of GAC conjugated to 8MTT , TT or CRM197. CD1 mice were immunized intraperitoneally (i.p.) at day 0 and 28 with 0.166, 0.5 and 1.5 μg GAC/dose with Alhydrogel 0.7 mg/mL (Al^{3+}). Summary graphs of anti-GAC specific IgG EU/mL reporting geometric means (bars) and individual antibody levels (dots).

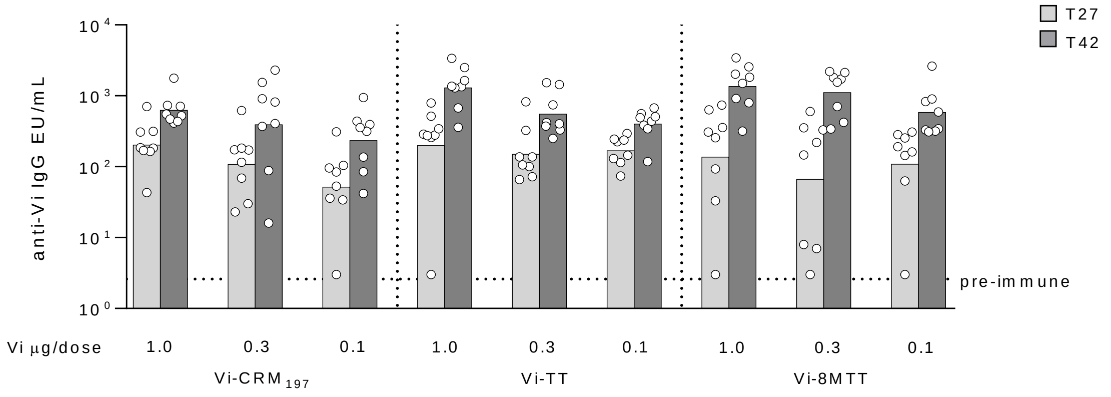

Figure S3. Immunogenicity in mice of Vi conjugated to 8MTT , TT or CRM197. CD1 mice were immunized intraperitoneally (i.p.) at day 0 and 28 with 0.1, 0.3 and 1.0 µg Vi/dose without Alhydrogel. Summary graphs of anti-Vi specific IgG EU/mL reporting geometric means (bars) and individual antibody levels (dots).

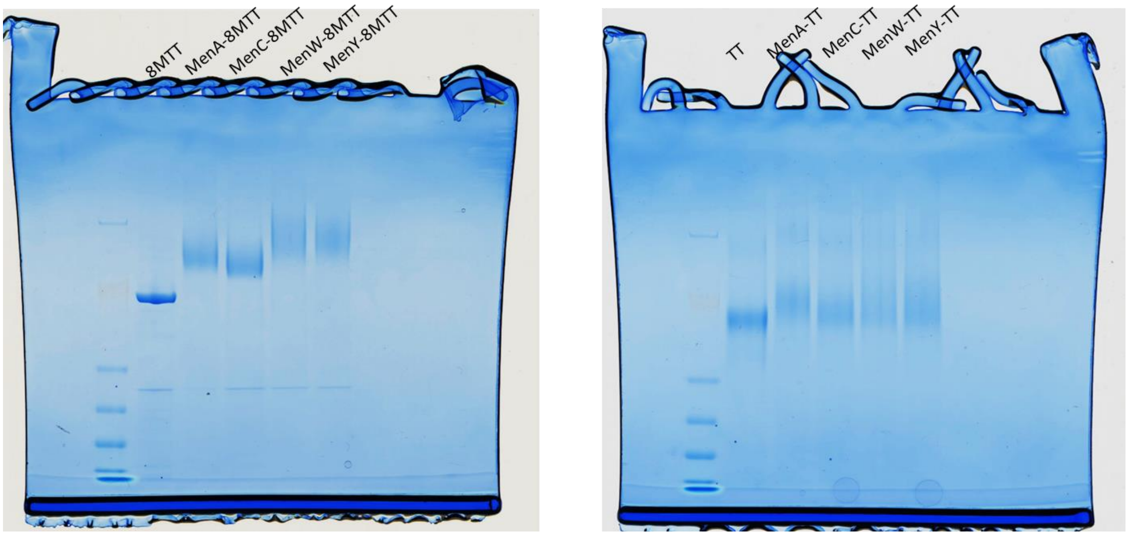

Figure S4. Characterization by SDS-PAGE analysis (7% Tris-acetate gel) of the Men glycoconjugates in comparison to unconjugated proteins. Original images of SDS-PAGE gels reported in Figure 2C.
